# Stage-associated enrichment of *Dietzia* in the conjunctival microbiome of calves with infectious bovine keratoconjunctivitis: a cross-sectional microbiome and genomic characterization study

**DOI:** 10.3389/fvets.2026.1890304

**Published:** 2026-07-16

**Authors:** Bolat Abdigulov, Anel Ordabayeva, Marat Kuibagarov, Alexandr Suminov, Oralbek Ilderbayev, Ayan Dauletov, Madina Kadyrova, Sarsenbay Abdrakhmanov, Gilles Vergnaud, Alexandr Shevtsov

**Affiliations:** 1 Laboratory of Applied Genetics, National Center of Biotechnology, Astana, Kazakhstan; 2 Department of General Biology and Genomics, L.N. Gumilyov Eurasian National University, Astana, Kazakhstan; 3 Center of Life Sciences, National Laboratory Astana, Nazarbayev University, Astana, Kazakhstan; 4 Institute of Animal Science and Veterinary, S. Seifullin Kazakh Agrotechnical University, Astana, Kazakhstan; 5 Institute for Integrative Biology of the Cell (I2BC), CEA, CNRS, Université Paris-Saclay, Gif-sur-Yvette, France

**Keywords:** 16S rRNA sequencing, cattle, conjunctival microbiome, *Dietzia*, infectious bovine keratoconjunctivitis, Kazakhstan, ocular microbiota, whole-genome sequencing

## Abstract

**Background:**

Infectious bovine keratoconjunctivitis (IBK) is a major ocular disease of cattle. Although the bovine conjunctival microbiome has been studied in IBK-affected and healthy animals, microbiome patterns across clinically defined lesion stages remain poorly understood, and the occurrence of *Dietzia* on the diseased bovine ocular surface has not been systematically investigated.

**Methods:**

This cross-sectional study enrolled 57 calves from two commercial farms in Kazakhstan, stratified across six clinical stages of IBK severity (0–5). Conjunctival swabs were analyzed by culture-based *Dietzia* recovery and 16S rRNA gene amplicon sequencing. Alpha and beta diversity were assessed using standard ecological metrics, and stage-associated *Dietzia* abundance was evaluated using Kruskal–Wallis testing and quadratic regression. Two representative *Dietzia* isolates were subjected to whole-genome sequencing, phylogenomic placement and homology-based screening against resistance and virulence-factor databases.

**Results:**

IBK stage was not associated with significant global restructuring of the conjunctival microbiome. Alpha-diversity indices did not differ significantly across stages, and PERMANOVA showed no stage-specific beta-diversity separation (*R*^2^ = 0.098, *p* = 0.323). In contrast, *Dietzia* showed a non-linear stage-associated abundance pattern in a quadratic model, with the highest reads-per-million (RPM) abundance in samples classified at stages 2–3, which are clinically characterized by active corneal ulceration. Culture-based recovery of *Dietzia* was frequent overall (42/57 samples; 73.7%) but showed no monotonic relationship with lesion severity. Whole-genome analysis of two isolates revealed phylogenomic heterogeneity: one isolate clustered within phylogenomic Group A, whereas the second was positioned outside the four major *Dietzia* groups, adjacent to the divergent animal isolate *Dietzia* sp. B32. Both genomes contained conserved stress-response, regulatory and metabolic homologues, without clear evidence of specialized virulence systems.

**Conclusion:**

IBK lesion stage was not associated with major alpha- or beta-diversity restructuring, but *Dietzia* displayed stage-associated enrichment during the active ulcerative phase. These findings support interpretation of bovine ocular *Dietzia* as a surface-accessible opportunistic colonizer rather than a confirmed primary aetiological agent of IBK.

## 1 Introduction

Infectious bovine keratoconjunctivitis (IBK), commonly referred to as pinkeye, is one of the most prevalent and economically important ocular diseases of cattle worldwide, occurring across major cattle-producing regions and production systems (1). The disease imposes substantial animal welfare and economic burdens through ocular pain, reduced weight gain in affected calves, decreased milk yield in dairy cattle, treatment and labour costs, and, in severe cases, permanent visual impairment or blindness. Although global prevalence has been estimated at approximately 2.78%, reported herd-level incidence varies widely, from less than 2% to more than 50%, depending on geographic region, season, breed composition, management system and husbandry practices. Available economic assessments, mainly from the United States, Australia and the United Kingdom, consistently indicate that IBK represents a significant financial burden for both beef and dairy industries (1–3). In Kazakhstan, IBK has also been documented in commercial cattle herds, including Aberdeen Angus and Kazakh White-headed breeds, with molecular evidence supporting a mixed-agent etiology (4–6).

The etiology of IBK is multifactorial. *Moraxella bovis*, a Gram-negative coccobacillus, is the best-established primary bacterial agent, with pathogenicity mediated through attachment pili and the RTX-family cytotoxin *MbxA*, facilitating corneal adhesion and epithelial damage (7, 8). Experimental challenge studies have consistently reproduced typical IBK lesions with *M. bovis*, whereas the contribution of the phylogenetically related *Moraxella bovoculi* remains unproven: experimental inoculation has not reproducibly induced classical corneal ulceration, and its detection in diseased eyes may reflect colonisation rather than primary causation (9, 10). *Mycoplasma bovoculi* has been identified as a potential predisposing or synergistic factor, based on its elevated prevalence in pre-outbreak and acute IBK herds (11). Bovine herpesvirus-1 may further predispose the ocular surface to bacterial colonisation through primary epithelial injury, while environmental factors including ultraviolet radiation, dust and face flies (*Musca autumnalis*) represent additional facilitating conditions (8, 12). Pathologically, IBK can progress from superficial epithelial disruption to corneal ulceration, perforation and permanent visual impairment, with breakdown of the epithelial barrier central to disease amplification (13).

The bovine ocular surface harbours a compositionally diverse resident microbiota that has only recently been characterized in depth using culture-independent high-throughput sequencing approaches. Previous studies have shown that the conjunctival microbiota of cattle commonly includes members of the phyla Proteobacteria, Bacillota/Firmicutes and Actinobacteria, with genera such as *Moraxella*, *Staphylococcus*, *Bacillu*s, *Paracoccus*, *Acinetobacter* and *Mycoplasma* frequently detected (14). Cullen et al. (15) demonstrated that the calf ocular microbiota differs between IBK-affected and healthy eyes at the community level, supporting the value of microbiome-based approaches as a complement to conventional diagnostics. Gafen et al. (16), analysing the conjunctival microbiome of 604 cattle eyes, reported taxon-level shifts in IBK-affected eyes, including changes involving Weeksellaceae, Methylobacteriaceae and Mycoplasmataceae, despite the absence of significant global alpha-diversity differences between affected and normal groups. Bartenslager et al. (17) longitudinally followed 227 pre-weaned calves and showed that ulcer presence did not strongly structure overall beta diversity, whereas temporal dynamics and animal-level factors contributed substantially to community variation. More recently, Kilama et al. (14) reviewed the bovine ocular microbiome and emphasised that ocular microbial communities are shaped by a complex interplay of host, herd, environmental, management and sampling-related factors. Together, these studies suggest that standard alpha- and beta-diversity metrics may be insufficient to resolve disease-associated microbial signals in a heterogeneous, low-biomass mucosal environment. Despite these advances, previous IBK microbiome studies have largely relied on binary comparisons between clinically affected and healthy animals. Whether the conjunctival microbiome differs across clinically defined IBK severity stages, from early serous conjunctivitis to corneal perforation, has not been systematically examined.

The genus *Dietzia* belongs to the family Dietziaceae, order Corynebacteriales, phylum *Actinomycetota*, and comprises Gram-positive, aerobic, non-acid-fast, mycolic acid-containing actinomycetes with high genomic GC content and considerable metabolic versatility. Since its establishment by Rainey et al. (18), the genus has expanded to include species recovered from diverse environments, including marine sediments, soils, hydrocarbon-contaminated substrates, aquatic habitats, food products and clinical specimens. Pan-genomic analyses indicate that the evolutionary diversification of *Dietzia* species is strongly influenced by ecological niche, with habitat-associated gene repertoires reflecting adaptation to different environmental conditions. Comparative genomic analysis of 56 *Dietzia* genomes by dos Santos et al. (19) identified four major phylogenomic groups or genomospecies and showed that traditional 16S rRNA-based species assignments do not always correspond to whole-genome relatedness. These findings support the use of whole genome-based approaches for taxonomic placement and ecological interpretation of disease-associated *Dietzia* isolates. Members of the genus may also be phenotypically similar to *Rhodococcus equi* and other mycolic acid-containing actinomycetes, which can lead to misidentification in clinical microbiology settings (20).

Although *Dietzia* is primarily known as an environmentally versatile actinomycete, several species have been reported as opportunistic pathogens or clinically relevant isolates in humans. Published clinical associations include bacteraemia in an immunocompromised patient (21), prosthetic joint infection (22), cutaneous and pulmonary infections, and confluent and reticulated papillomatosis (23). Of particular relevance to ocular disease, a case of post-traumatic endophthalmitis following penetrating injury with a retained metallic foreign body has been attributed to *Dietzia* (24), suggesting that members of the genus may occasionally access or colonise ocular tissues when anatomical barriers are severely disrupted. However, such reports do not establish *Dietzia* as a primary ocular pathogen in livestock. Rather, the environmental persistence and metabolic versatility of *Dietzia*—including a pan-genome enriched in lipid metabolism genes, the capacity of some species to utilise structurally diverse carbon substrates, including fatty acids and hydrocarbons, and a mycolic acid-containing cell envelope associated with tolerance to physicochemical stress—provide a plausible ecological framework for understanding how these bacteria may persist in nutrient-variable, host-associated niches (19, 25–27). By extension, disruption of the corneal epithelial barrier during IBK progression could transiently increase the availability of host-derived nutrients, including lipid- and protein-rich material. Such conditions may favour opportunistic taxa with broad catabolic repertoires; however, for *Dietzia* in the bovine ocular surface this remains an ecological hypothesis rather than a demonstrated pathogenic mechanism (28, 29).

Despite the growing literature on the bovine ocular microbiome and on *Dietzia* as an opportunistic actinomycete, the abundance dynamics, culture-based recovery patterns and genomic characteristics of *Dietzia* across clinically defined IBK lesion stages have not been investigated. It remains unclear whether *Dietzia* represents a stable commensal member of the bovine conjunctival microbiota, a stage-associated opportunistic colonizer linked to corneal tissue damage, or a microorganism whose clinical relevance in bovine ocular disease remains unresolved. Addressing this question requires an integrated approach combining clinical severity stratification, culture-based isolation, community-level microbiome profiling and whole-genome characterization of representative isolates—an approach not previously applied to *Dietzia* in the context of veterinary ocular infectious disease.

The present study was therefore designed to: (i) characterize the conjunctival microbiome of IBK-affected and clinically healthy calves across six clinically defined lesion stages using *16S rRNA* gene amplicon sequencing and community-level diversity analyses; (ii) assess culture-based recovery of *Dietzia* across the clinical severity spectrum of IBK; (iii) determine whether *Dietzia* abundance follows a stage-associated, non-linear pattern using normalized read counts and quadratic regression modelling; and (iv) contextualize two representative cultured bovine ocular *Dietzia* isolates within the available genomic diversity of the genus using whole-genome sequencing, Average Nucleotide Identity (ANI)-based phylogenomic placement, and in silico screening of resistance- and putative virulence-associated gene homologues against the CARD, VFDB and VICTORS databases. It should be noted that the present study was not designed to identify the primary aetiological agent of IBK; rather, it aimed to characterize the stage-associated abundance dynamics and genomic features of *Dietzia* as a member of the diseased bovine conjunctival microbiome.

## 2 Materials and methods

### 2.1 Study design, animals and clinical grading

This observational cross-sectional study with severity-stratified sampling was conducted to characterize the clinical and microbiological features of infectious bovine keratoconjunctivitis (IBK) in calves aged 4–6 months. Animals of two breeds (Aberdeen Angus and Kazakh White-headed) from two commercial farms were examined under field conditions at a single time point. Clinical severity at the time of sampling was used as a categorical surrogate for disease progression, under the assumption that stages 0–5 represent sequential pathogenetic phases of IBK; causal inferences regarding temporal dynamics are therefore limited by the cross-sectional nature of the design.

Calves presenting with clinical signs consistent with IBK—including lacrimation, blepharospasm, photophobia, and/or corneal lesions of any severity—were enrolled. Clinically healthy calves without ocular abnormalities were included as controls (stage 0). Animals with ocular trauma, non-infectious ocular diseases, or systemic conditions potentially affecting ocular health were excluded.

Each eye was scored individually using a six-point clinical scale (stages 0–5), based on visual examination under natural daylight and 1% sodium fluorescein staining. Excess dye was rinsed with sterile saline, and corneal assessment was performed within 30–60 s.

The scoring system reflects the sequential pathogenic progression of IBK and integrates three parameters: depth of tissue involvement (epithelium → stroma → intraocular structures), ulcer extent (expressed as fractions of the corneal surface), and reactive changes (neovascularization, fibrinous exudation, uveal prolapse). Classification was assigned according to the most severe clinical feature observed.

**Figure 1:**
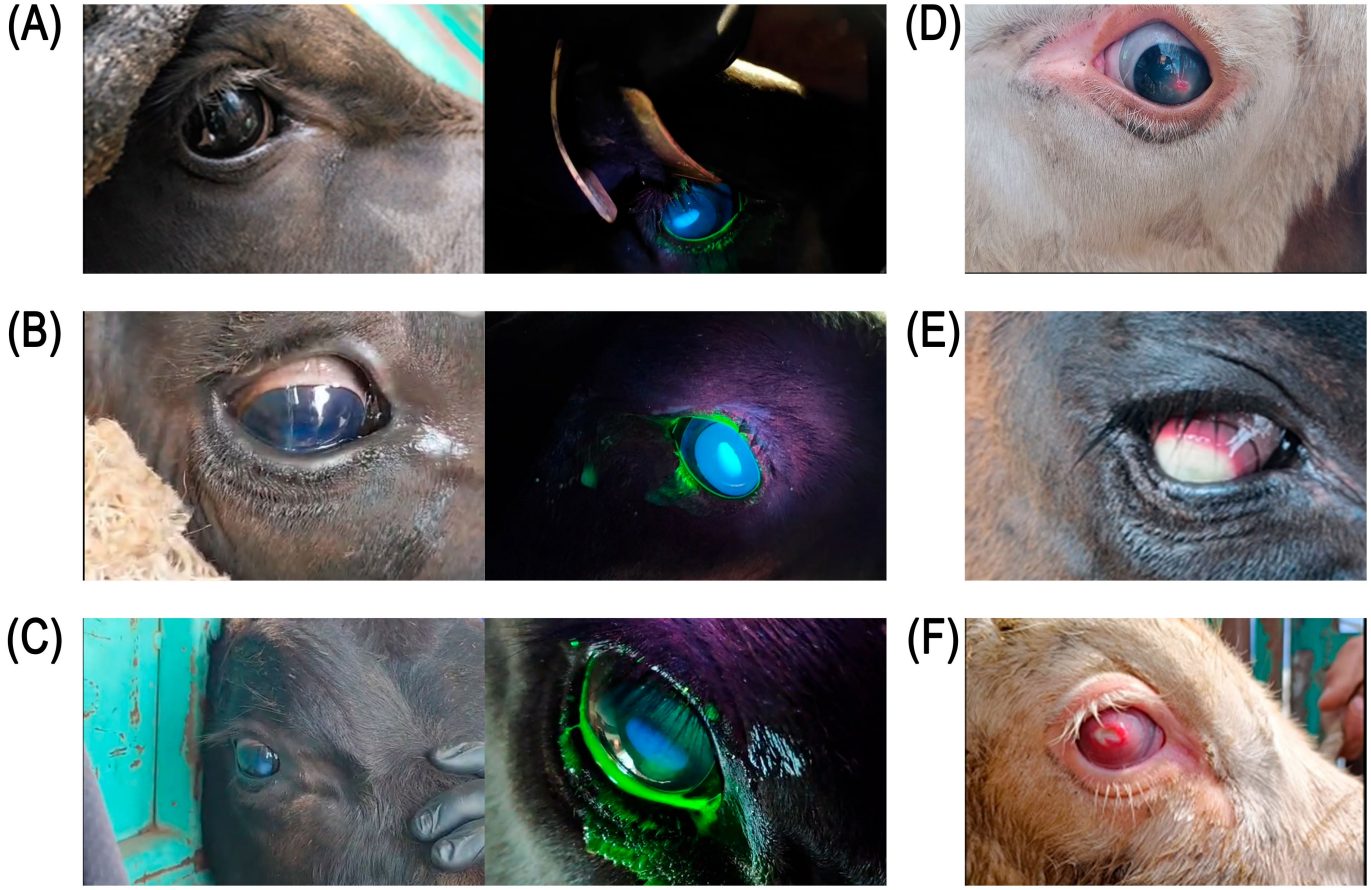
Representative ocular photographs illustrating the six-stage clinical severity classification of infectious bovine keratoconjunctivitis in calves: **(A)** Stage 0 — control; **(B)** Stage 1 — serous conjunctivitis; **(C)** Stage 2 — superficial ulcerative keratitis; **(D)** Stage 3 — stromal keratitis with early neovascularization; **(E)** Stage 4 — severe keratouveitis; **(F)** Stage 5 — corneal perforation.

Stage 0—Control. Clear, reflective cornea; non-injected conjunctiva; absence of lacrimation, blepharospasm, and photophobia; fluorescein-negative (Figure 1A).

Stage 1—Serous conjunctivitis without corneal involvement. Lacrimation, blepharospasm, photophobia, and conjunctival hyperaemia; cornea remains transparent; fluorescein-negative (Figure 1B). Represents functional irritation without structural corneal damage.

Stage 2—Superficial ulcerative keratitis. Localized epithelial defect appearing as a grey-white lesion, central or paracentral, involving <1/3 of the corneal surface; fluorescein-positive with sharply demarcated bright-green uptake; surrounding cornea remains clear, without neovascularization (Figure 1C).

Stage 3—Stromal keratitis with early neovascularization. Diffuse greyish-blue corneal opacity due to stromal edema and cellular infiltration; ulcer extends over 1/3–2/3 of the cornea; superficial limbal vessels impart a pinkish hue (early pannus formation) (Figure 1D). This stage marks the transition from superficial to deep keratitis.

Stage 4—Severe keratouveitis. Ulcerative-necrotic lesions involve >2/3 of the cornea, which becomes markedly opaque with both superficial and deep neovascularization (established pannus); fibrinous or fibrinopurulent exudate is present in the anterior chamber (including possible hypopyon), resulting in a yellowish appearance; pupillary response is reduced or absent (Figure 1E).

Stage 5—Corneal perforation (terminal stage). Full-thickness corneal defect with iris prolapse, appearing as a dark-red or pigmented tissue mass protruding through the cornea; descemetocele formation or leakage of intraocular contents may be observed. This stage is associated with loss of globe integrity and typically irreversible vision loss (Figure 1F).

All procedures were conducted in accordance with relevant institutional and national guidelines for animal care and use. The study protocol was approved by the Local Ethics Committee of the National Center for Biotechnology of the Republic of Kazakhstan (Protocol No. 4, dated 24 October 2023).

### 2.2 Sample collection

Ocular samples were collected prior to fluorescein instillation to avoid any potential effect of the dye on bacterial viability and downstream microbiome analysis. One eye per animal was sampled. In animals with unilateral or asymmetric ocular involvement, the eye exhibiting the more severe clinical stage was selected for sampling. In clinically healthy control animals (stage 0), the right eye was sampled by default. Following manual restraint of the head and gentle retraction of the lower eyelid, sterile cotton-tipped swabs were rolled across the corneal surface and adjacent bulbar conjunctiva, while avoiding contact with the eyelid margins and eyelashes.

Two swabs were collected sequentially from the same eye: the first swab was intended for bacterial culture and was immediately placed into Amies transport medium, whereas the second swab was designated for microbiome analysis and was placed into a sterile dry tube without transport medium. No treatment was administered prior to or during sample collection. Following completion of sampling, IBK-affected animals received appropriate veterinary treatment in accordance with standard practice and animal welfare requirements.

Samples were transported to the laboratory in a cool box at +2 to +8 °C within 8 h of collection. Upon arrival, swabs in Amies medium were processed immediately for bacterial culture, while dry swabs were stored at −80 °C until DNA extraction for microbiome analysis.

### 2.3 Bacterial isolation and species identification

Swabs preserved in Amies transport medium were resuspended in 1 mL of sterile 0.9% NaCl by vortexing to release adherent microorganisms. A 50 μL aliquot of the resulting suspension was spread onto Columbia Agar Base (EP, USP, ISO; cat. no. 1104, Condalab, Madrid, Spain) supplemented with 7% (v/v) defibrinated horse serum (cat. no. HOS-1A, Capricorn Scientific, Ebsdorfergrund, Germany), and incubated aerobically at 37 °C for 48 h. Following incubation, each plate was divided into 4 or 6 sectors, adjusted so that each sector contained between 10 and 20 colonies. All colonies within one designated sector were subcultured to ensure representative recovery of the dominant microbiota. In addition, any colony exhibiting a morphology or pigmentation distinct from those present in the selected sector was subcultured independently, regardless of its location on the plate. All selected isolates were sub-cultured twice on the same medium to obtain pure cultures. Other microorganisms were also isolated and identified from conjunctival samples across clinical stages; however, as the present study was specifically focused on *Dietzia,* their further characterisation and analysis were intentionally outside the scope of this work.

Genomic DNA was extracted from pure cultures using the QIAamp DNA Mini Kit (Qiagen, Hilden, Germany) according to the manufacturer’s instructions. Species identification was performed by partial *16S rRNA* gene sequencing using universal bacterial primers 8F (5′-AGAGTTTGATCCTGGCTCAG-3′) and 806R (5′-GGACTACCAGGGTATCTAAT-3′) (30). Sanger sequencing was carried out using the BigDye Terminator v3.1 Cycle Sequencing Kit (Applied Biosystems, Vilnius, Lithuania) on an ABI 3730xl DNA Analyzer (Applied Biosystems/Hitachi, Tokyo, Japan). Raw sequences were manually inspected and trimmed prior to analysis. Taxonomic assignment was performed by querying sequences against the NCBI GenBank (nr/nt) database using BLASTn. Isolates were assigned to the genus level as *Dietzia* spp. based on partial *16S rRNA* gene sequencing; species-level assignments based on 16S rRNA were considered provisional and were not used for definitive taxonomic conclusions.

### 2.4 Whole-genome sequencing and analysis

Genomic DNA extracted from pure cultures was quantified using the Qubit dsDNA HS and BR Assay Kits on a Qubit 2.0 Fluorometer (Invitrogen/Life Technologies, Carlsbad, CA, USA). Sequencing libraries were prepared from 50 ng of input DNA using the Collibri™ ES DNA Library Prep Kit for Illumina Systems with Combinatorial Dual Indexes (cat. no. A38605024; Thermo Fisher Scientific, Waltham, MA, USA), according to the manufacturer’s instructions. Libraries were purified with the kit-supplied magnetic beads, amplified using the Collibri™ Library Amplification Master Mix, and quantified using the Qubit dsDNA HS Assay Kit. Library purity was assessed with a NanoDrop spectrophotometer (Thermo Fisher Scientific). Paired-end sequencing (2 × 300 bp) was performed on an Illumina MiSeq platform using the MiSeq Reagent Kit v3, 600 cycles (cat. no. MS-102-3003; Illumina, San Diego, CA, USA).

### 2.5 16S rRNA gene amplicon sequencing and microbiome profiling of the ocular surface

Total genomic DNA was extracted from dry swabs (stored at −80 °C) using the QIAamp DNA Mini Kit (Qiagen, Hilden, Germany) according to the manufacturer’s protocol, including an enzymatic pre-lysis step with lysozyme to ensure efficient disruption of Gram-positive cell walls. DNA concentration and purity were assessed with a Qubit 2.0 Fluorometer (Invitrogen, Carlsbad, CA, USA) and a NanoDrop spectrophotometer (Thermo Fisher Scientific, Waltham, MA, USA); a reagent-only extraction blank was processed in parallel as a negative control.

Amplicon libraries for microbiocenosis analysis were prepared following the Illumina 16S Metagenomic Sequencing Library Preparation protocol (31). The V3–V4 hypervariable regions of the *16S rRNA* gene were amplified using universal primers described by Klindworth et al. (32) with Illumina overhang adapter sequences (Forward: 5′-TCGTCGGCAGCGTCAGATGTGTATAAGAGACAGCCTACGGGNGGCWGCAG-3′; Reverse: 5′-GTCTCGTGGGCTCGGAGATGTGTATAAGAGACAGGACTACHVGGGTATCTAATCC-3′). PCR amplification was performed in a total volume of 25 μL using Phusion High-Fidelity DNA Polymerase with HF buffer (Thermo Scientific Baltics UAB, Vilnius, Lithuania). Amplicons were purified using AMPure XP beads (Beckman Coulter, Beverly, MA, USA), followed by index incorporation using AmpliSeq CD Indexes for Illumina (Illumina, San Diego, CA, USA). The resulting libraries were normalized, pooled, and sequenced on the Illumina MiSeq platform using the MiSeq Reagent Kit v3 (2 × 300 bp).

Raw paired-end *16S rRNA* gene sequences were subjected to quality control, and reads were processed for adapter removal using Cutadapt (v2.6) (33). Adapter sequences were trimmed from both the 3′ and 5′ ends, and only reads in which adapters were detected were retained. Quality-trimmed reads were aligned against the SILVA 138.2 *16S rRNA* reference database (34) using KMA (v1.4.9) (35), which maps reads directly against redundant databases via k-mer seeding and Needleman–Wunsch extension. Alignments were filtered to a minimum template coverage of 30% and a minimum query identity of 90%. Per-template depth was normalized to reads per million (RPM) using total mapped reads extracted from each sample’s*.mapstat* file.

Taxonomic entries matching the string “*Dietzia*” (case-insensitive) were extracted from each sample’s quality-filtered result file. Where multiple matching templates passed the quality thresholds, the single entry with the highest alignment depth was retained per sample, preventing double-counting of closely related reference sequences. Per-sample *Dietzia* RPM values were merged with the sample metadata table to link each observation to its disease stage.

Non-parametric approaches were used throughout, as abundance data were non-normally distributed. Differences in *Dietzia* RPM across the six disease stages (Stages 0–5) were assessed using the Kruskal–Wallis rank-sum test. Significant overall effects were followed by pairwise Dunn’s post-hoc tests with Bonferroni correction for multiple comparisons, implemented via the *rstatix* R package (36).

To investigate whether *Dietzia* abundance follows a non-linear trajectory across disease progression, a quadratic regression model was fitted to log-transformed abundance values (log₁₊[RPM]). Disease stage was encoded as a continuous numeric variable (0–5), and the model included both a linear (Stage) and a quadratic (Stage^2^) term:


log1+(RPM)∼β0+β1×Stage+β2×Stage2+ε

The significance of each coefficient was assessed from the ordinary least-squares model summary. A statistically significant negative *β*₂ coefficient would indicate an inverted U-shaped (humped) relationship, consistent with peak *Dietzia* abundance at intermediate disease stages followed by a decline at advanced stages. Model fit was visualized by overlaying the predicted quadratic curve with 95% confidence intervals onto per-stage boxplots of log₁₊(RPM).

Alpha diversity (Shannon H′, Simpson 1 − D, observed richness) was computed per sample from the full RPM community matrix using the *vegan* R package (v2.6) (37) and compared across stages by Kruskal–Wallis and Dunn’s post-hoc tests with Bonferroni correction. Beta diversity was assessed via Bray–Curtis dissimilarities, visualized by principal coordinates analysis (PCoA), and tested by PERMANOVA (*adonis2*, 999 permutations) (38). Homogeneity of multivariate group dispersion was evaluated using *betadisper* as a complementary diagnostic to PERMANOVA. All analyses were performed in R (≥ 4.3).

### 2.6 Bioinformatic analysis of WGS

Raw paired-end sequencing reads were assessed for quality using FastQC (v0.11.9) (39). Adapter trimming and quality filtering were performed with Trimmomatic (v0.40) (40) in paired-end mode. Post-trimming quality was verified with a second FastQC run.

Trimmed paired-end reads were assembled *de novo* using SPAdes (v4.2.0) (41) with the—careful flag to minimize assembly errors, which is recommended for small bacterial genomes. The resulting assemblies were evaluated with QUAST (v5.2.0) (42). Scaffolds generated by SPAdes served as input for all downstream analyses.

Genome annotation was carried out using Prokka (v1.14.6) (43) with taxonomy set to *Dietzia* sp. and the kingdom parameter set to Bacteria. The—rfam flag was enabled for non-coding RNA prediction, and the—compliant flag was used to ensure compatibility with NCBI submission standards.

Screening for virulence-associated genes and antibiotic resistance determinants was performed using ABRicate (v1.0.1) (44) against two databases: VFDB (Virulence Factor Database) (45) and VICTORS (46). Both databases were queried with a minimum nucleotide identity threshold of 70% and a minimum coverage of 60%, consistent with thresholds applied in comparative genomics studies of actinobacteria. For antibiotic resistance gene identification, the CARD (Comprehensive Antibiotic Resistance Database) (47) was additionally queried using the same identity and coverage thresholds. Two genome assemblies (scaffolds in FASTA format) were screened independently, and results were summarized using the ABRicate summary function to generate a presence/absence matrix across strains. Genes with hits below the identity threshold were excluded from downstream interpretation.

Sequenced genomes were positioned within *Dietzia* sp. using the ANI approach. ANI values were calculated using FastANI version 1.34 (48). We included in the analysis the 56 assemblies previously investigated (19). We updated this collection by searching for *Dietzia* in NCBI and in the Genome Taxonomy Database (GTDB) release 11-RS232 (15th April 2026) (49). Publicly available WGS data including assemblies and short read archives (SRA) data were recovered from NCBI https://www.ncbi.nlm.nih.gov/ or EBI-ENA https://www.ebi.ac.uk/ena. The FastANI matrix was imported into BioNumerics version 8.1 (Applied-Maths, Sint-Martens-Latem, Belgium) for cluster analysis and graph production. The BioNeighbor Joining algorithm was used (50).

## 3 Results

### 3.1 Culture-based recovery of *Dietzia* isolates across clinical stages

*Dietzia* isolates were recovered during year 2025 from ocular samples across all clinical stages, including clinically normal eyes. Overall, *Dietzia* was isolated from 42 of 57 examined samples, corresponding to a culture-based recovery rate of 73.7% (Table 1). The recovery rate varied among clinical stages, from 57.1% at stage 1 to 90.9% at stage 2, without a strictly monotonic increase or decrease across lesion severity. The recovery rate in the control group (71.4%) is similar to the other groups. Thus, culture-based recovery of *Dietzia* was not directly proportional to clinical severity.

**Table 1:** Culture-based recovery of *Dietzia* isolates according to clinical stage of ocular lesion.

Clinical stage	Samples examined, *n*	*Dietzia*-positive samples, *n*	Recovery rate, % (95% CI)
0	7	5	71.4 (35.9–91.8)
1	7	4	57.1 (25.0–84.2)
2	11	10	90.9 (62.3–98.4)
3	13	8	61.5 (35.5–82.3)
4	11	9	81.8 (52.3–94.9)
5	8	6	75.0 (40.9–92.9)
Total	57	42	73.7 (61.0–83.4)

Importantly, these data represent the proportion of samples yielding cultivable *Dietzia* isolates and should not be interpreted as quantitative estimates of bacterial load or within-sample abundance. When multiple colonies assigned to *Dietzia* were recovered from the same sample, only one representative isolate was retained for downstream identification and analysis. Therefore, Table 1 reflects sample-level recovery of cultivable *Dietzia*, rather than the true numerical abundance of *Dietzia* within ocular samples.

### 3.2 Overall conjunctival microbiome diversity across clinical stages

**Figure 2:**
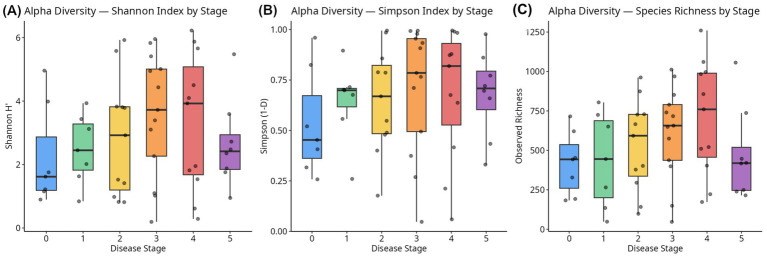
Alpha diversity of conjunctival microbiome in calves with infectious bovine keratoconjunctivitis stratified by clinical stage. **(A)** Shannon index (H′); **(B)** Simpson index (1 − D); **(C)** observed species richness. Boxplots show the median, interquartile range, and 1.5 × IQR whiskers; dots represent individual samples.

Alpha diversity metrics—Shannon index (H′), Simpson index (1 − D), and observed species richness—were calculated for each clinical stage (Figure 2). All three indices showed a similar non-monotonic trend across stages 0–5, with a general increase from early to intermediate stages and a subsequent decline at the final stage.

The Shannon index increased from a mean of 2.23 ± 1.59 at stage 0 (control group) to a peak of 3.57 ± 1.93 at stage 3, followed by a moderate decrease at stage 4 (3.32 ± 2.16) and a more pronounced decline at stage 5 (2.65 ± 1.38). A comparable pattern was observed for the Simpson index, which rose from 0.53 ± 0.26 at stage 0 to 0.71 ± 0.31 at stage 3, remaining relatively stable through stage 5 (0.68 ± 0.21). Observed species richness similarly increased from a mean of 419.3 ± 202.6 at stage 0 to a maximum of 704.2 ± 360.9 at stage 4, with a subsequent decrease to 472.6 ± 289.7 at stage 5.

Although none of the three diversity metrics reached statistical significance across stages (Kruskal–Wallis test: Shannon *H* = 3.24, *p* = 0.663; Simpson *H* = 3.00, *p* = 0.699; observed richness *H* = 5.63, *p* = 0.344), numerical differences were observed, with higher mean values at intermediate stages (3, 4) and lower values at stages 0–1 and 5. However, within-group variance was substantial at all stages, particularly stages 3 and 4, and the current sample size may have been insufficient to detect stage-associated differences in alpha-diversity metrics if such differences exist. These results indicate that no statistically detectable stage-associated differences in alpha diversity were observed in the present cohort.

**Figure 3:**
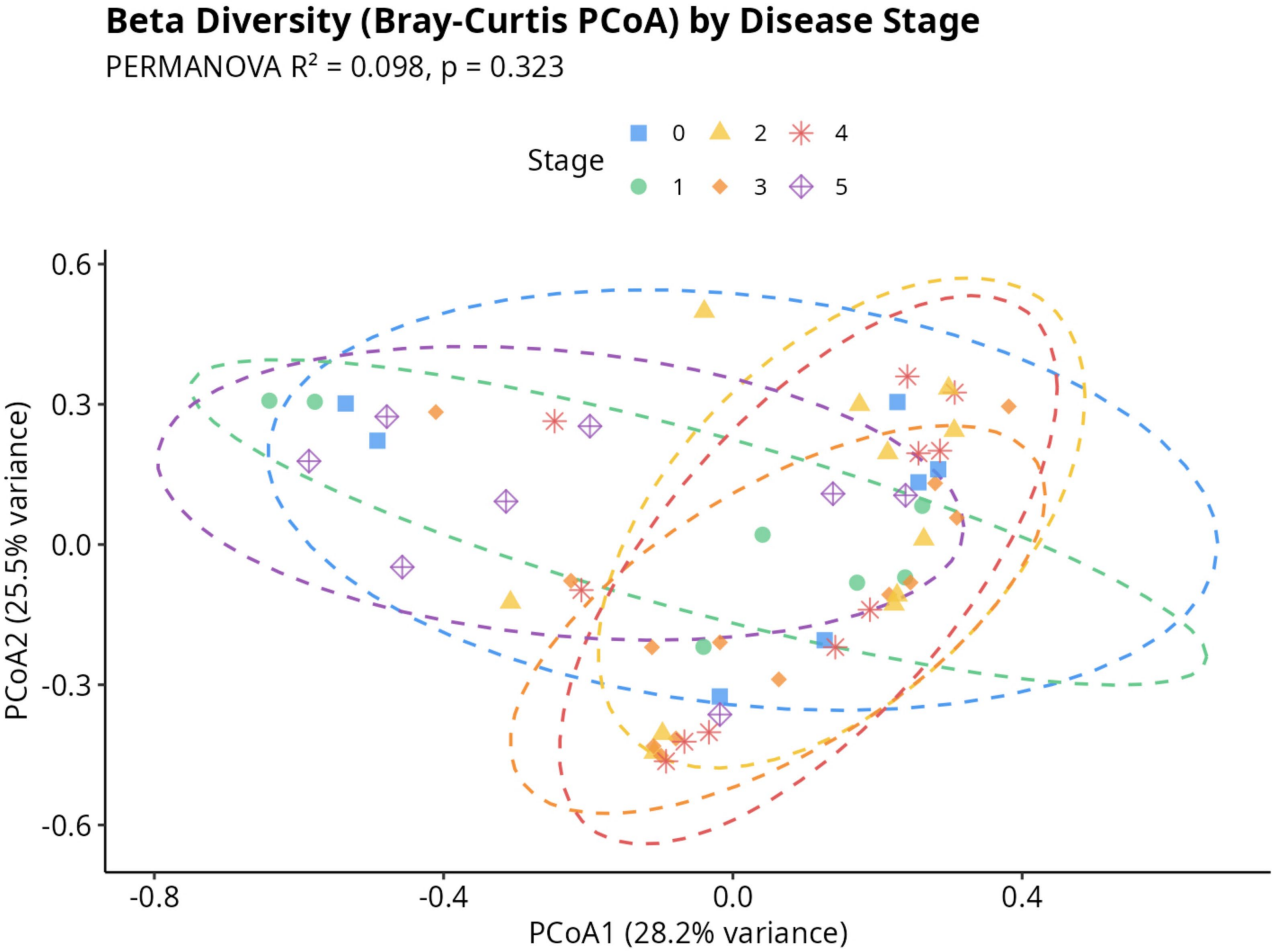
Beta diversity (Bray–Curtis PCoA) of conjunctival microbiome in calves with infectious bovine keratoconjunctivitis by disease stage. Dashed ellipses represent 95% confidence regions per group. PERMANOVA: *R*^2^ = 0.098, *p* = 0.323.

**Figure 4:**
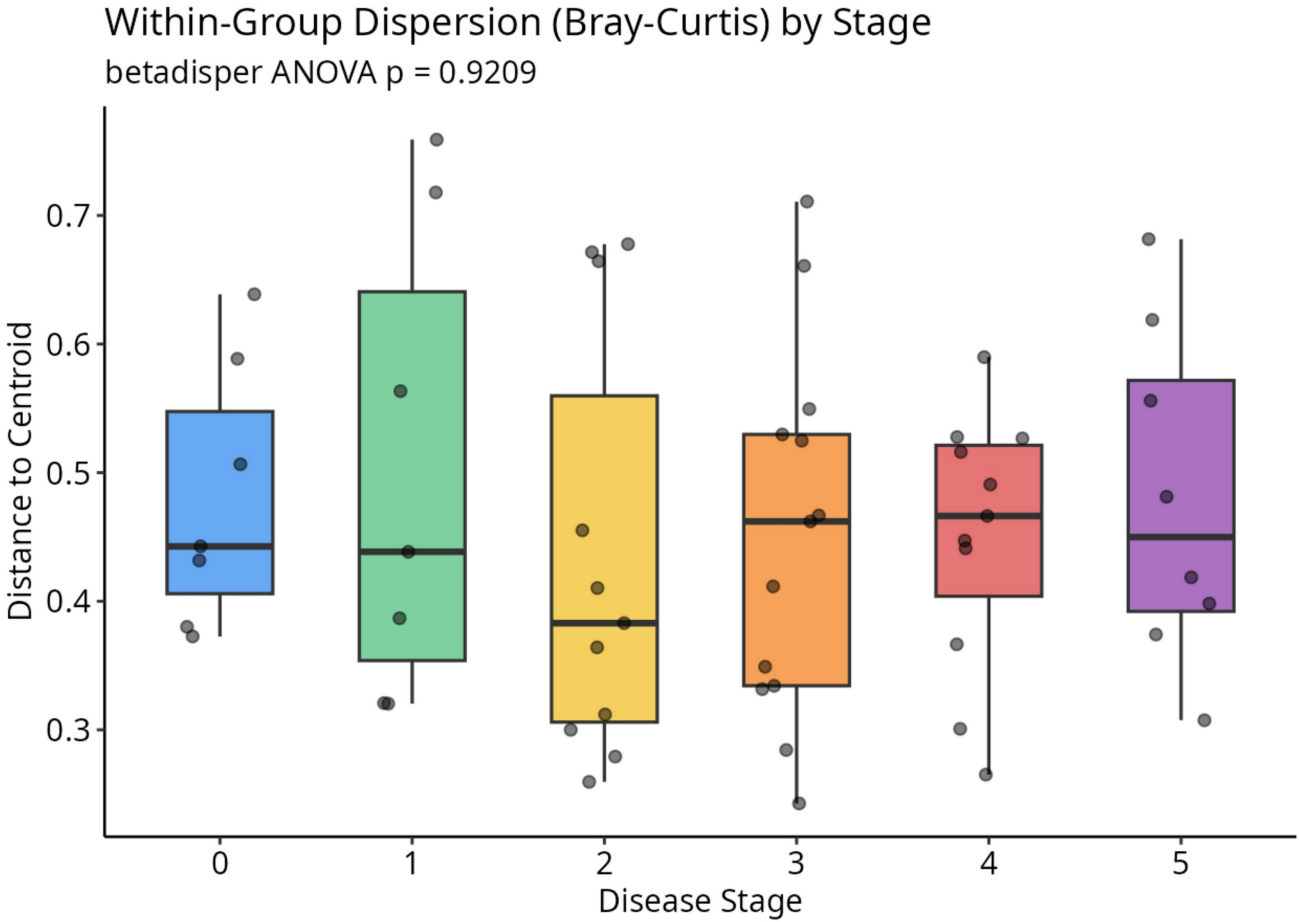
Within-group Bray–Curtis dispersion (distance to group centroid) by disease stage. Betadisper ANOVA *p* = 0.921, indicating homogeneous multivariate dispersion across stages.

To assess differences in overall community composition among clinical stages, Bray–Curtis dissimilarities were visualized by PCoA and tested using PERMANOVA (Figure 3). Homogeneity of multivariate dispersion was evaluated using betadisper (Figure 4).

The PCoA ordination (Figure 3) revealed substantial overlap between the confidence ellipses of all six stages along both principal coordinates (PCoA1: 28.2% variance explained; PCoA2: 25.5% variance). No stage-specific clustering was evident, with samples from all groups distributed broadly across the ordination space. PERMANOVA confirmed the absence of a statistically significant effect of disease stage on overall community composition (*R*^2^ = 0.098, *p* = 0.323, df = 5), indicating that disease stage accounts for approximately 9.8% of the total variation in Bray–Curtis distances, but this effect did not reach significance at the *α* = 0.05 threshold given the current sample size.

Assessment of within-group multivariate dispersion (betadisper) revealed comparable centroid distances across all six stages (ANOVA F (5,51) = 0.282, *p* = 0.921; Figure 4), confirming that the PERMANOVA assumption of homogeneous dispersion was met. Taken together, the beta diversity analyses indicate that the overall compositional structure of the conjunctival microbiome does not significantly differ across IBK severity stages (PERMANOVA *R*^2^ = 0.098, *p* = 0.323).

### 3.3 Clinical-stage-associated abundance pattern of *Dietzia*

**Figure 5:**
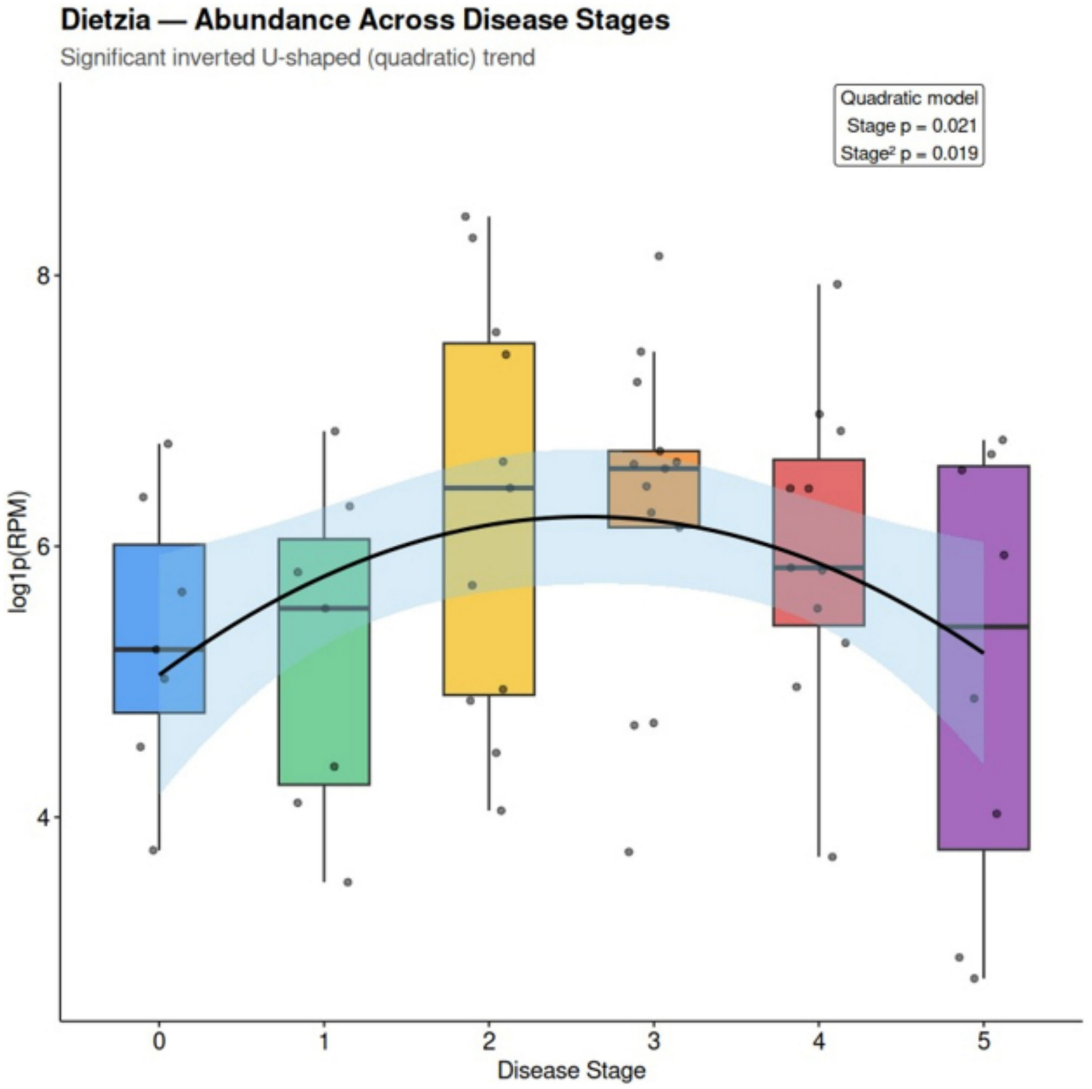
Clinical-stage-associated abundance pattern of *Dietzia* in conjunctival swabs from calves with infectious bovine keratoconjunctivitis. Boxplots show log1p-transformed RPM values by clinical stage; dots represent individual samples. The fitted quadratic regression curve with 95% confidence interval is overlaid. The model showed significant linear and quadratic terms, consistent with a non-linear abundance pattern with higher *Dietzia* abundance at stages 2–3.

RPM abundance of the genus *Dietzia* was detected in all samples across all six clinical stages (prevalence 100%; *n* = 57). The median RPM values were 187.3 (stage 0), 254.1 (stage 1), 619.3 (stage 2), 715.1 (stage 3), 343.5 (stage 4), and 253.6 (stage 5). On the log1p (RPM) scale, this corresponds to median values of 5.24, 5.54, 6.43, 6.57, 5.84, and 5.54 for stages 0–5, respectively, consistent with an inverted U-shaped (quadratic) trend. Kruskal–Wallis test on raw RPM values did not reach statistical significance (abundance: *p* = 0.291; coverage depth: *p* = 0.384), reflecting high within-group variability; however, a quadratic regression model on log1p (RPM) values identified a significant quadratic association between clinical stage and log-transformed *Dietzia* abundance (linear term: *p* = 0.021; quadratic term: *p* = 0.019), consistent with higher *Dietzia* abundance at stages 2–3 with a decline at both early and late stages (Figure 5).

The highest *Dietzia* abundance at stages 2–3 corresponds to active corneal ulceration (from <1/3 to 1/3–2/3 of the corneal surface) accompanied by stromal opacity and early limbal neovascularization. At advanced stages (4, 5), characterized by extensive corneal involvement, fibrinous keratouveitis, and, in some cases, perforation, *Dietzia* abundance decreased to levels comparable to early-stage or control conditions, suggesting that the peak abundance is associated with the active inflammatory phase of disease rather than its sequelae.

### 3.4 Whole-genome sequencing and assembly of two representative isolates

Two isolates were selected for whole-genome sequencing to represent opposite ends of the IBK clinical spectrum: IBK-p2-15D (stage 1, Kazakh White-headed) and IBK-p2-16D (stage 5, Aberdeen Angus).

Illumina MiSeq sequencing generated ~5.1 to 5.6 million paired-end reads per isolate, corresponding to 1.3–1.4 Gbp of data (Table 2). The GC content of raw reads (70–71%) was consistent with high-GC Actinomycetota genomes.

**Table 2:** Summary of sequencing output and de novo assembly statistics for the two sequenced isolates.

Metric	IBK-p2-15D	IBK-p2-16D
Host breed	Kazakh Whiteheaded	Aberdeen Angus
Clinical stage	Stage 1	Stage 5
Raw reads	5,087,020	5,555,536
Total bases	1.3 Gbp	1.4 Gbp
Raw GC (%)	71	70
Contigs (≥ 500 bp)	51	31
Contigs ≥ 50 kbp	15	10
Total assembly length (bp)	3,556,514	3,681,159
Largest contig (bp)	594,428	942,033
N50 (bp)	232,102	409,305
N75 (bp)	149,776	323,212
L50	4	3
L75	9	5
GC (%)	70.58	70.45
Ambiguous bases (Ns per 100 kbp)	10.8	8.04
Modal coverage (×)	~128	~120

*De novo* assembly produced draft genomes of comparable size and GC content, with assembly statistics consistent with sufficient quality for comparative genomic analysis. The assembly of IBK-p2-16D was more contiguous, with fewer contigs and higher N50 values compared to IBK-p2-15D, indicating improved assembly continuity. In both isolates, the majority of the genome was contained within a limited number of large contigs (≥50 kbp), and the proportion of ambiguous bases was low, supporting overall assembly completeness and quality.

Assembly GC content closely matched that of the raw reads, suggesting the absence of substantial contamination. Sequencing depth was high (~120–130×) and uniform across both genomes, with no evidence of secondary coverage modes that would indicate mixed populations or assembly artefacts.

### 3.5 Phylogenomic placement of the Kazakh isolates in the global *Dietzia* context

**Figure 6:**
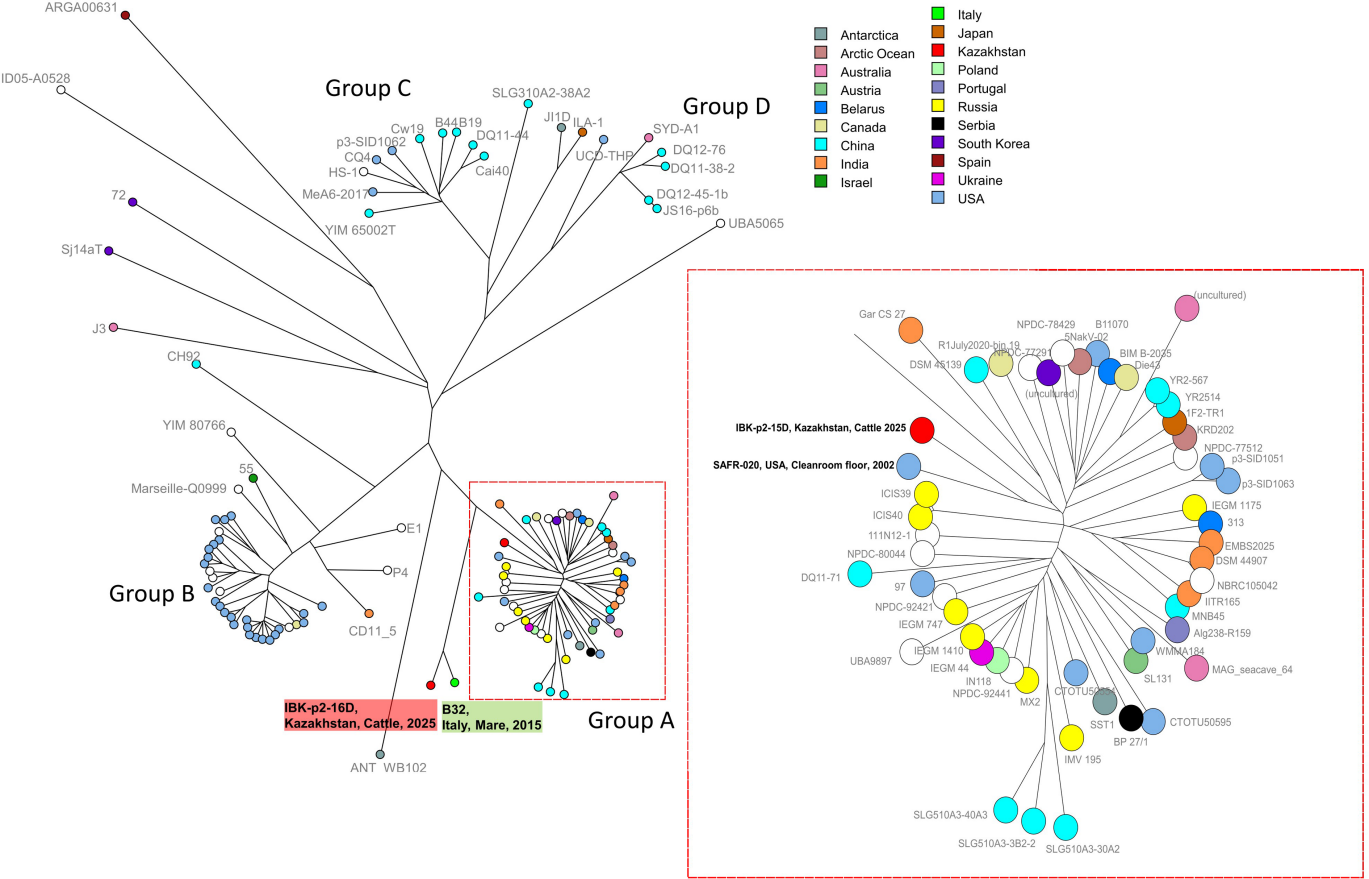
Phylogenomic placement of two Kazakh bovine ocular Dietzia isolates within the genus *Dietzia*. An unrooted phylogenomic tree was constructed using ANI genomic distances and includes a total of 119 genomes spanning the known diversity of the genus *Dietzia*. Clade designations A–D are shown in accordance with the phylogenomic framework of (19).

Phylogenomic placement of the two Kazakh bovine ocular *Dietzia* isolates was assessed using ANI-based genomic distance analysis against publicly available genomes of the genus *Dietzia*. A total of 140 WGS datasets (assemblies and sequence read archives) were identified (Supplementary Table S1), including the 56 datasets previously investigated (19). Twenty-three were duplicates (same biosample accession, or identical strain Id or alias and ANI value above 98.5 with a monophyletic clustering). Group assignments of the 117 unique datasets and of IBK-p2-15D and IBK-p2-16D reported here were interpreted according to the framework of dos Santos et al. (19), who defined four major phylogenomic groups, or genomospecies, designated A–D by using whole-genome similarity, ANI, dDDH and pangenome approaches (Figure 6; Supplementary Table S2).

IBK-p2-15D was positioned within Group A comprising a total of 53 entries (Figure 6). In the dos Santos et al. framework, Group A comprised 18 genomes assigned to *D. maris*, *D. kunjamensis*, *D. kunjamensis* subsp. *schimae* and *D. alimentaria*, with reported ANI values >95%, dDDH values >70% and a pangenome *α* value of 0.74. In the enlarged view of Group A, IBK-p2-15D was located closest to *Dietzia* sp. SAFR-020, an isolate recovered from a cleanroom floor in the USA (pairwise ANI value, 97.5%, Supplementary Table S2).

In contrast, IBK-p2-16D was positioned outside Groups A–D and formed a separate branch adjacent to *Dietzia* sp. B32 with a pairwise ANI value of 98.4%. *Dietzia* sp. B32 was recovered from diffuse subcutaneous nodules of a mare in Reggio Calabria, Italy, and was reported as the first *Dietzia* genome obtained from an animal source (19). B32 did not show ANI values above 95% with the other genomes analyzed by dos Santos et al., supporting its distinct genomospecies status within the genus.

Overall, the two Kazakh bovine ocular isolates occupied distinct phylogenomic positions, with a pairwise ANI value of 90.4% (Supplementary Table S2). IBK-p2-15D belongs to Group (genomospecies) A, whereas IBK-p2-16D was placed outside the four major groups defined by dos Santos et al., defining a new candidate genomospecies with strain B32 from Italy.

### 3.6 Predicted resistome and virulence-associated gene content

The draft genomes of IBK-p2-15D and IBK-p2-16D were screened against CARD, VFDB and VICTORS using minimum thresholds of ≥70% nucleotide identity and ≥60% query coverage. Because these databases contain reference loci from characterized bacterial pathogens, the detected genes should be considered homology-based predictions rather than experimentally validated virulence or resistance determinants in the analyzed *Dietzia* isolates. Full per-hit statistics, including alignment coordinates, coverage, identity and database annotations, are provided in Supplementary Tables S3–S5.

CARD screening identified two rifamycin resistance-associated loci in both isolates: *rpoB2*, encoding a rifampin-resistant RNA polymerase *β*-subunit paralogue, and *RbpA*, encoding an RNA polymerase-binding protein (Tables 3, 4).

**Table 3:** Summary of resistance and virulence-associated gene screening in two *Dietzia* isolates.

Database	IBK-p2-15D	IBK-p2-16D	Shared loci	Isolate-specific loci
CARD	2	2	2	None
VFDB	10	11	10	*panC* only in IBK-p2-16D
VICTORS	53	52	47*	15D: *xthA, ung, ceoB, ceoC, gcvT, scpA*; 16D: *furB, gcp, mutY, panC, rimI*

**Table 4:** Main functional groups of predicted resistance and virulence-associated homologues.

Functional group	Representative genes	Main products / functions
Rifamycin-associated resistance	*rpoB2, RbpA*	RNA polymerase β-subunit paralogue; RNA polymerase-binding protein
Oxidative and stress response	*ahpC, sodA, dnaK*	Peroxide detoxification; superoxide detoxification; chaperone response
Regulation	*sigA/rpoV, sigE, sigH, mprA, mtrA, regX, relA, crp, kstR*	Sigma factors, two-component regulators, stringent-response and global transcriptional regulators
DNA repair / genome maintenance	*recA, xthA, ung, mutY, gcp/rimI*	Recombination and base-excision/DNA-damage repair
Metabolic adaptation	*icl, narH, panD, panC, coaA, ino1*	Carbon, nitrate, pantothenate/coenzyme A and cell-envelope precursor metabolism
Lipid / cell envelope functions	*fadA6, fabG, echA20, ltp2/ltp3, accD1/scoB, yrbE1B/yrbE4A, rfbE*	Lipid metabolism, envelope-associated transport and surface-related functions

VFDB screening identified 10 virulence-associated homologues in IBK-p2-15D and 11 in IBK-p2-16D. Ten loci were shared by both isolates and were assigned to three major functional categories: oxidative and stress response (*ahpC, sodA*), transcriptional and stress regulation (*sigA/rpoV, sigE, sigH, mprA, relA*), and metabolic adaptation (*icl, narH, panD*). One additional gene, *panC*, encoding pantothenate synthetase, was detected only in IBK-p2-16D.

VICTORS screening returned 53 database hits in IBK-p2-15D and 52 in IBK-p2-16D. After collapsing entries mapping to the same genomic locus, the dataset comprised 58 non-redundant loci, including 47 shared loci, six loci detected only in IBK-p2-15D, and five loci detected only in IBK-p2-16D. Shared loci covered several functional groups. These included oxidative and protein-stress response (*ahpC, sodA, dnaK*), regulatory systems (*mtrA, mprA, regX, sigE, sigH, crp, kstR*), and DNA repair and genome maintenance (*recA*). Additional shared loci spanned central and intermediary metabolism (*icl, ino1, aceE, coaA, glpX, ilvB1/ilvH, leuD, lysA, panD*), lipid and cell-envelope-associated functions (f*adA6, fabG, echA20, ltp2, ltp3, desA3, accD1/scoB, yrbE1B, yrbE4A*), and cell-wall or cell-division-related genes (*ddl, ftsY, rfbE*).

The IBK-p2-15D-specific VICTORS loci included *xthA, ung, ceoB, ceoC, gcvT* and *scpA*. The IBK-p2-16D-specific loci included *furB, gcp, mutY, panC* and *rimI*. Thus, the two isolates showed a largely overlapping repertoire of predicted virulence-associated homologues, with strain-specific differences restricted to a small subset of DNA-repair, regulatory, transport and metabolic genes.

## 4 Discussion

In the present study, the conjunctival microbiome of calves with infectious bovine keratoconjunctivitis (IBK) was evaluated in relation to clinical disease severity, and the microbiome analysis was complemented by whole-genome sequencing of two cultured *Dietzia* isolates recovered from clinically distinct stages. By stratifying samples according to clinically defined lesion stages, this cross-sectional study enabled comparison of ocular microbiome patterns across the clinical spectrum of IBK, rather than relying solely on a binary affected-versus-unaffected framework used in previous studies (15–17). In this context, the key finding was not a global restructuring of the conjunctival microbiome—alpha- and beta-diversity did not significantly differ between stages—but a stage-associated, taxon-specific signal: the genus *Dietzia* showed a non-linear increase in relative abundance, peaking during the active ulcerative phase of IBK.

At the community level, IBK lesion stage was not associated with a detectable global restructuring of the conjunctival microbiome in this cohort. Alpha-diversity indices showed numerical stage-associated variation but did not differ significantly among clinical stages, and Bray–Curtis-based PCoA and PERMANOVA did not reveal significant stage-specific clustering. Homogeneous multivariate dispersion further supported the validity of the PERMANOVA interpretation. These findings limit conclusions at the whole-community level, although they do not exclude taxon-specific changes. Similar observations have been reported in previous bovine ocular microbiome studies, where disease status or ulcer presence did not consistently produce strong alpha- or beta-diversity separation, whereas taxon-level shifts and host-, herd-, environmental-, management- and sampling-related factors contributed to community variation (14, 16, 17, 51, 52). This background heterogeneity may limit the ability of global alpha- and beta-diversity metrics to resolve disease-stage-associated changes, highlighting the importance of complementary taxon-level analyses.

Against this globally non-stratified community background, *Dietzia* showed a distinct taxon-specific pattern, with an inverted U-shaped abundance trajectory peaking at stages 2–3. Although Kruskal–Wallis testing on raw RPM values did not reach significance, quadratic regression of log-transformed abundance identified a significant non-linear association with disease stage. Thus, the main microbiome signal in this study was not global dysbiosis, but stage-associated genus-level enrichment of *Dietzia* during the active ulcerative phase of IBK.

This pattern is consistent with a limited, niche-driven opportunistic response to local tissue damage. Disruption of the corneal epithelial barrier (53) may transiently increase the availability of host-derived substrates (28), conditions consistent with opportunistic enrichment of bacteria capable of environmental persistence and metabolic flexibility, a profile well documented for mycolic acid-containing actinomycetes such as *Dietzia* (18, 19, 25, 27, 54). The decline at stages 4–5 may reflect altered late-stage surface conditions, competitive replacement, or reduced surface accessibility; however, these explanations remain hypothetical because host inflammatory mediators, tear-film chemistry, and microbial activity were not directly assessed.

Although *Dietzia* species have been reported from opportunistic human infections and clinical specimens (29, 55, 56), including a case of post-traumatic endophthalmitis following penetrating ocular injury (24), the present data do not demonstrate tissue invasion or a primary aetiological role of *Dietzia* in IBK. In this cohort, *Dietzia* may therefore be more appropriately interpreted as a stage-associated, surface-accessible opportunistic member of the diseased ocular microbiome, with enrichment during the active ulcerative phase rather than evidence of causation.

Whole-genome sequencing of two representative Dietzia isolates provided genomic context for the microbiome findings. The isolates were recovered from clinically distinct stages and different farm backgrounds, one from early disease and one from terminal disease. Both genomes showed features consistent with high-GC *Actinomycetota* (57) and were assembled as draft genomes of comparable size and sequencing depth.

The phylogenomic analysis showed that *Dietzia* isolates recovered from the ocular surface of IBK-affected cattle in this study occupied distinct phylogenomic positions, rather than forming a single clonal group. IBK-p2-15D was affiliated with Group A, a broad and genomically coherent lineage that includes multiple currently named *Dietzia* taxa. This placement indicates that at least one bovine ocular isolate falls within a broadly represented *Dietzia* lineage that includes genomes from diverse environmental and host-associated sources.

IBK-p2-16D showed a different pattern, being positioned outside the four major genomospecies and close to *Dietzia* sp. B32. Because B32 was previously described as an animal-derived and genomically separate isolate, the proximity of IBK-p2-16D to this branch is notable. However, this observation should be interpreted cautiously, because only two bovine ocular genomes were analyzed.

The contrasting positions of IBK-p2-15D and IBK-p2-16D indicate phylogenomic heterogeneity among bovine ocular *Dietzia* isolates. This finding is consistent with the broader genomic complexity of the genus reported by dos Santos et al. (19), who showed that traditional species assignments within *Dietzia* do not always correspond to whole-genome relatedness. Therefore, genome-based analysis provides a more appropriate framework for contextualizing disease-associated *Dietzia* isolates than *16S rRNA*-based identification alone.

Importantly, the present dataset does not allow a direct causal association between phylogenomic placement and IBK severity. The two sequenced isolates originated from different clinical stages and different farm backgrounds, but the sample size is insufficient to determine whether genomic lineage, accessory gene content, host factors or farm-level exposures contribute to disease progression. Additional bovine ocular *Dietzia* genomes from multiple farms and clinical stages will be required to assess whether specific phylogenomic lineages are enriched in severe IBK lesions. Importantly, we show here that genetically very distinct Dietzia strains seem to be normal constituents of the ocular microbiome.

Screening of the two bovine ocular *Dietzia* genomes against CARD, VFDB and VICTORS revealed a restricted set of resistance-associated homologues and a broader set of homologues of genes annotated as virulence-associated in pathogen-focused databases. Because VFDB and VICTORS are enriched for genes characterized in established pathogens, particularly *Mycobacterium tuberculosis*, their detection in *Dietzia* should be interpreted as homology to pathogen-associated database entries, not as direct evidence of bona fide virulence. This distinction is especially relevant for actinobacteria, where genome-based classification has shown that whole-genome phylogenies provide substantially higher resolution than single-gene approaches and better resolve relationships among closely related Corynebacteriales lineages (58). Accordingly, many of the homologues detected in *Dietzia* may reflect shared actinobacterial ancestry rather than pathogenic specialization, and are more conservatively interpreted as conserved functions involved in stress tolerance, metabolic flexibility and environmental persistence.

The resistome of both isolates was limited to two rifamycin-associated determinants, *rpoB2* and *RbpA*, with no additional CARD hits. In the absence of phenotypic antimicrobial susceptibility testing, these CARD hits should be interpreted as resistance-associated homologues rather than confirmed resistance phenotypes. Notably, *rpoB2* has previously been identified among resistance-associated loci in comparative genomic analysis of the *Dietzia* core genome (19). Experimental evidence from *Nocardia* further supports the role of *rpoB2* as a rifampin-resistance determinant, as *rpoB* duplication correlated with rifampin resistance, transfer of *rpoB2* conferred resistance to a susceptible strain, and deletion of *rpoB2* abolished significant resistance (59). *RbpA*, in turn, is an actinobacterial RNA polymerase-binding transcription factor associated with the principal RNA polymerase holoenzyme and basal rifampicin tolerance (60).

Among the virulence-associated homologues, the oxidative-stress defence genes *ahpC* and *sodA* are the most relevant to the IBK context. *AhpC* encodes an alkyl hydroperoxide reductase involved in protection against reactive oxygen and nitrogen intermediates; in *M. tuberculosis*, disruption of *ahpC* increases susceptibility to peroxynitrite and reduces survival in unstimulated macrophages, supporting its role in antioxidant defence during host-associated stress (61). *SodA* encodes an iron-cofactored superoxide dismutase that catalyzes the conversion of superoxide radicals into hydrogen peroxide and molecular oxygen. In *M. tuberculosis*, reduced *SodA* production increased susceptibility to hydrogen peroxide and resulted in marked attenuation in mice, supporting the role of iron-cofactored SOD in oxidative-stress defence and host-associated survival (62). Detection of *ahpC* and *sodA* in both *Dietzia* isolates therefore does not imply tissue-invasive pathogenicity, but indicates a potential capacity to tolerate oxidative stress on the inflamed ocular surface.

The presence of *sigE, sigH, mprA* and *relA* further indicates conserved stress-response regulatory circuitry. In *M. tuberculosis*, *SigE* contributes to resistance against heat, surface and oxidative stress and is required for optimal survival in macrophage models (63), whereas *SigH* regulates major components of the oxidative and heat-stress response, including thioredoxin/thioredoxin reductase and heat-shock genes, and also influences *sigE* expression (64). The two-component regulator *MprA* has been implicated in persistent infection in *M. tuberculosis* (65), and experimental work in mycobacteria supports a regulatory link between *MprAB, SigE* and *Rel*, connecting stress sensing with activation of the stringent-response pathway (66). More generally, (p)ppGpp-mediated stringent responses coordinate bacterial adaptation to nutrient limitation and other stresses and are linked to persistence and stress tolerance (67). Therefore, the detection of homologues of these regulatory components in *Dietzia* is consistent with conserved actinomycete stress-adaptation capacity, but their expression and functional contribution to ocular persistence require experimental validation.

Metabolic homologues further support an opportunistic-survival model. Isocitrate lyase (ICL), encoded by icl, is a key enzyme of the glyoxylate shunt, which enables carbon assimilation from acetyl-CoA generated during fatty acid *β*-oxidation. In *Mycobacterium tuberculosis*, disruption of icl attenuates persistence in macrophages and mice without substantially affecting acute-phase growth (68), and ICL1 and ICL2 are jointly required for fatty acid metabolism and *in vivo* virulence (69). These data support the broader relevance of glyoxylate-shunt-associated metabolism during host adaptation, although the functional role of icl in *Dietzia* remains to be experimentally tested. The *panD/panC* pair encodes consecutive steps in pantothenate biosynthesis, a precursor pathway for coenzyme A. The exclusive detection of *panC* in IBK-p2-16D represents one of the few differences between the isolates and should be interpreted cautiously given the limited number of genomes analyzed.

The broader VICTORS profile reinforces the opportunistic colonizer interpretation. Shared loci included genes associated with DNA repair and recombination (*recA*), protein homeostasis (*dnaK*), two-component and transcriptional regulation (*mtrA, mprA, regX3*), lipid and cell-envelope functions (*fadA6, fabG, echA20, accD1, ltp2/ltp3*), MCE-family transport (*yrbE1B, yrbE4A*) and cofactor biosynthesis (*coaA, ino1*). In *M. tuberculosis*, representative members of these functional groups have been linked to stress adaptation, host-associated metabolism and persistence-related phenotypes (70–75). However, given the phylogenetic distance between *Dietzia* and obligate intracellular pathogens, these homologues are more conservatively interpreted as indicators of conserved actinobacterial regulatory, metabolic and envelope-associated capacity rather than as direct evidence of specialized pathogenicity.

No database hits corresponding to major specialized virulence systems, such as type III or type IV secretion systems, pore-forming toxins or high-copy surface-protein virulence families, were detected in either genome. Although draft assemblies may miss fragmented loci, the high sequencing depth and assembly continuity reduce the likelihood that the absence of multiple major virulence-system signatures is solely an assembly artefact.

The strain-specific VICTORS loci are most parsimoniously interpreted as strain-level genomic variation rather than stage-dependent adaptation. IBK-p2-15D carried *xthA, ung, ceoB/ceoC, gcvT* and *scpA*, whereas IBK-p2-16D carried *furB, gcp/rimI, mutY* and *panC*. These genes are associated with DNA repair, metal/redox regulation, transport and metabolism. The non-overlapping DNA-repair loci are notable, but whether they reflect lineage history, environmental selection or assembly-related differences cannot be resolved from the present dataset.

Overall, the microbiome and genomic findings support an interpretation of bovine ocular *Dietzia* as a stage-associated opportunistic colonizer of the inflamed or ulcerated ocular surface or as a normal constituent of the ocular microbiome, rather than as a confirmed primary aetiological agent of IBK. This interpretation is consistent with the observed increase in *Dietzia* abundance during intermediate ulcerative stages and with the genomic profile of the sequenced isolates, which was dominated by conserved stress-response, regulatory and metabolic homologues rather than specialized virulence systems. Future studies including additional bovine ocular *Dietzia* genomes, phenotypic antimicrobial susceptibility testing, oxidative-stress survival assays and transcriptomic analyses under ocular inflammation-like conditions will be required to determine which of the predicted loci are expressed and functionally relevant during host association.

This study has several limitations. First, the cross-sectional design does not allow temporal or causal inference, and clinical stage was used as a categorical surrogate for disease progression rather than being observed longitudinally within the same animals. Second, stratification of 57 calves across six clinical stages necessarily results in small within-stage sample sizes (ranging from 7 to 13 animals per group), which reduces the statistical power of between-stage comparisons, increases susceptibility to inter-group data fluctuation, and limits the ability to detect subtle community-level differences that may exist across the clinical spectrum of IBK. Third, *16S rRNA* gene profiling provides genus-level resolution for *Dietzia* and does not determine whether the detected reads correspond to viable, metabolically active or clinically relevant populations; culture-independent abundance estimates may also be influenced by DNA from non-viable cells. Fourth, only two cultured *Dietzia* isolates were subjected to whole-genome sequencing at draft level, precluding generalisation of phylogenomic placement or accessory gene content and preventing any direct link between genomic features, IBK severity and farm-level exposures. Fifth, animals were sourced from two farms of different breeds, and farm-level and breed-level effects on microbiome composition could not be separated from disease-stage effects. Furthermore, resistance- and virulence-associated genes were inferred from database homology alone and were not validated by phenotypic antimicrobial susceptibility testing, oxidative-stress assays, transcriptomics or experimental infection models. In addition, the culture-based protocol was not designed for quantitative CFU enumeration of *Dietzia* at each disease stage; future studies incorporating quantitative culture methods would allow direct comparison of bacterial load with microbiome-based abundance estimates and clinical severity. Finally, bacterial culturing was performed under aerobic conditions only, in line with the focus on recovering and characterizing Dietzia as an aerobic actinomycete. Therefore, the culture-based results should not be interpreted as a complete inventory of viable IBK-associated microorganisms, and obligate anaerobes potentially present in inflammatory or necrotic ocular lesions could not be detected.

## 5 Conclusion

This study shows that clinically defined IBK lesion stages were not associated with a detectable global restructuring of the conjunctival microbiome at the alpha- or beta-diversity level in this cohort. However, *Dietzia* displayed a stage-associated, non-linear abundance pattern, with higher RPM values during intermediate ulcerative stages and lower abundance in early/control and advanced lesions. Culture-based recovery confirmed that cultivable *Dietzia* was present across all clinical stages, including the healthy control population, with an overall recovery rate of 73.7% and without a strictly monotonic relationship with disease severity. Whole-genome analysis of two isolates revealed phylogenomic heterogeneity and a gene repertoire dominated by conserved stress-response, regulatory and metabolic homologues rather than specialized virulence systems. Taken together, these findings support the interpretation of bovine ocular *Dietzia* as a normal constituent or a surface-accessible opportunistic member of the diseased ocular microbiome, enriched during the active ulcerative phase in this dataset, rather than as a confirmed primary aetiological agent of IBK.

## Data Availability

All data from this project are publicly available on NCBI. The draft genome assemblies generated in this study have been deposited in NCBI GenBank under accession numbers JBYMTK000000000 and JBYMTL000000000.

## Supplementary material

SUPPLEMENTARY TABLE S1 List of 140 publicly available Dietzia sp. WGS datasets evaluated in this investigation.

SUPPLEMENTARY TABLE S2 Metadata and pairwise ANI values of 119 non-redundant WGS datasets, including the two datasets produced in this report.

SUPPLEMENTARY TABLE S3 CARD antibiotic resistance determinants.

SUPPLEMENTARY TABLE S4 VFDB virulence factor homologues.xlsx.

SUPPLEMENTARY TABLE S5 VICTORS broader virulence-associated homologues.
